# First person – Clare Muir

**DOI:** 10.1242/dmm.052588

**Published:** 2025-08-29

## Abstract

First Person is a series of interviews with the first authors of a selection of papers published in Disease Models & Mechanisms, helping researchers promote themselves alongside their papers. Clare Muir is first author on ‘ A subset of neutrophil phagosomes is characterised by pulses of Class I PI3K activity’, published in DMM. Clare conducted the research described in this article while a Wellcome 4ward North Clinical Academic Fellow in Professor Steven Renshaw and Professor Alison Condliffe's lab at Department of Infection, Immunity & Cardiovascular Disease, University of Sheffield, UK. Clare is now a Senior Clinical Lecturer in Veterinary Anatomic Pathology at the University of Edinburgh, investigating how neutrophils and macrophages kill and digest prey.


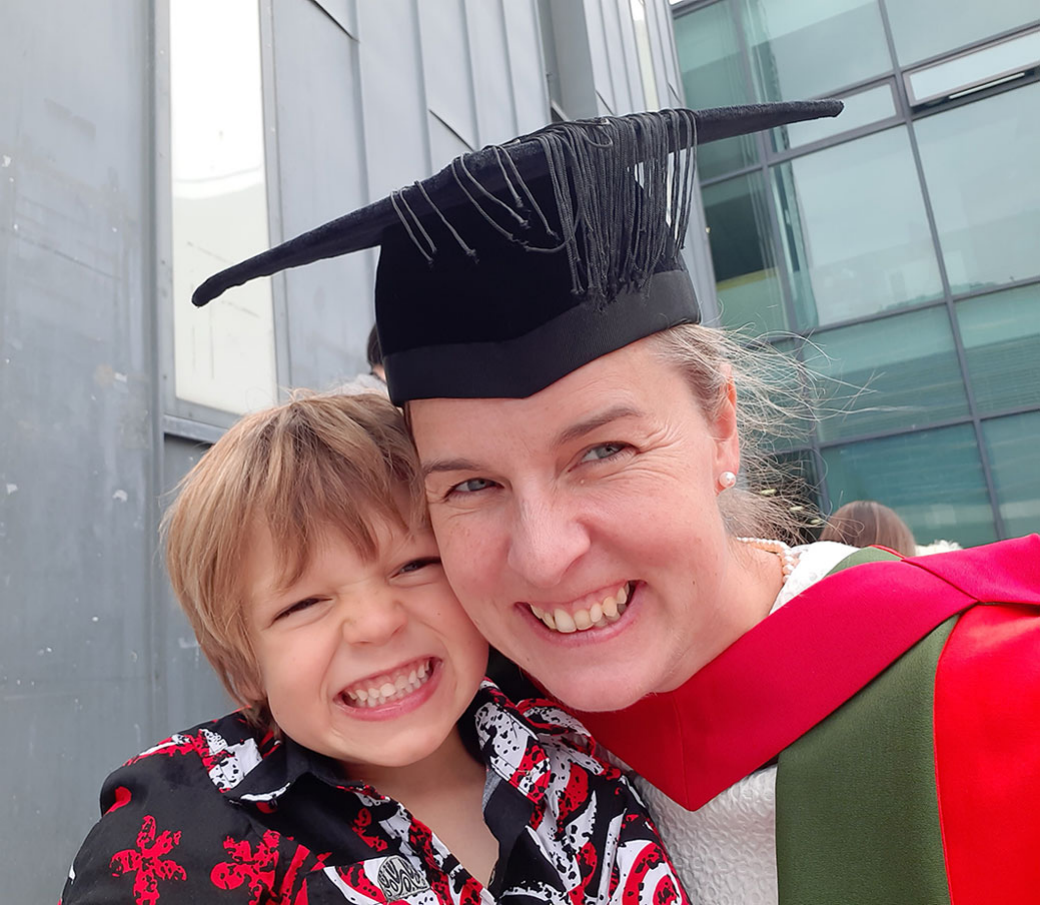


**Clare Muir**

**Who or what inspired you to become a scientist?**

I became interested in research whilst training as a veterinary pathologist. I was curious to understand the mechanisms that caused disease in the animals that had been presented to the clinic. I remember diagnosing a lysosomal storage disease in a young cat and Professor Cox offering me the opportunity to work the case up at the University of Cambridge. I really enjoyed the experience of working in a laboratory and decided to do a PhD.

I became interested in research whilst training as a veterinary pathologist.

**What is the main question or challenge in disease biology you are addressing in this paper?**

This project started following an observation by Felix Ellett who was currently working as a postdoc in Professor Renshaw's lab. Felix noticed that a reporter for some of the lipids that form the phagosome membrane [PIP3 and PI(3,4)P2] re-recruited in pulsatile bursts to a subset of neutrophil phagosomes. This was unexpected as PIP3 and PI(3,4)P2 typically diminish from phagosomes shortly after closure. I spent about 18 months optimising high detail imaging of neutrophil phagosomes that had formed during a bacterial skin infection of a living zebrafish and identified that these pulses followed repeated reopening/closure of a subset of neutrophil phagosomes.

**How would you explain the main findings of your paper to non-scientific family and friends?**

Neutrophils are a type of immune cell that eats, kills and digests bacteria in membrane-bound vesicles called phagosomes. This paper describes that neutrophils leave some phagosomes open. We are not sure what the purpose of having leaky phagosomes is; however, we think it may enable neutrophils to tell other cells what they have eaten and therefore optimise the immune response.

**What are the potential implications of these results for disease biology and the possible impact on patients?**

Leaky phagosomes could have a positive or a negative impact on inflammation resolution. Excessive release of phagosome content could contribute to inflammation and may enable live bacteria to escape/proliferate within tissue. However, regulated release of phagosome contents could enable neighboring cells to adapt their response and make the immune response more effective at killing the pathogen.

**Figure:**
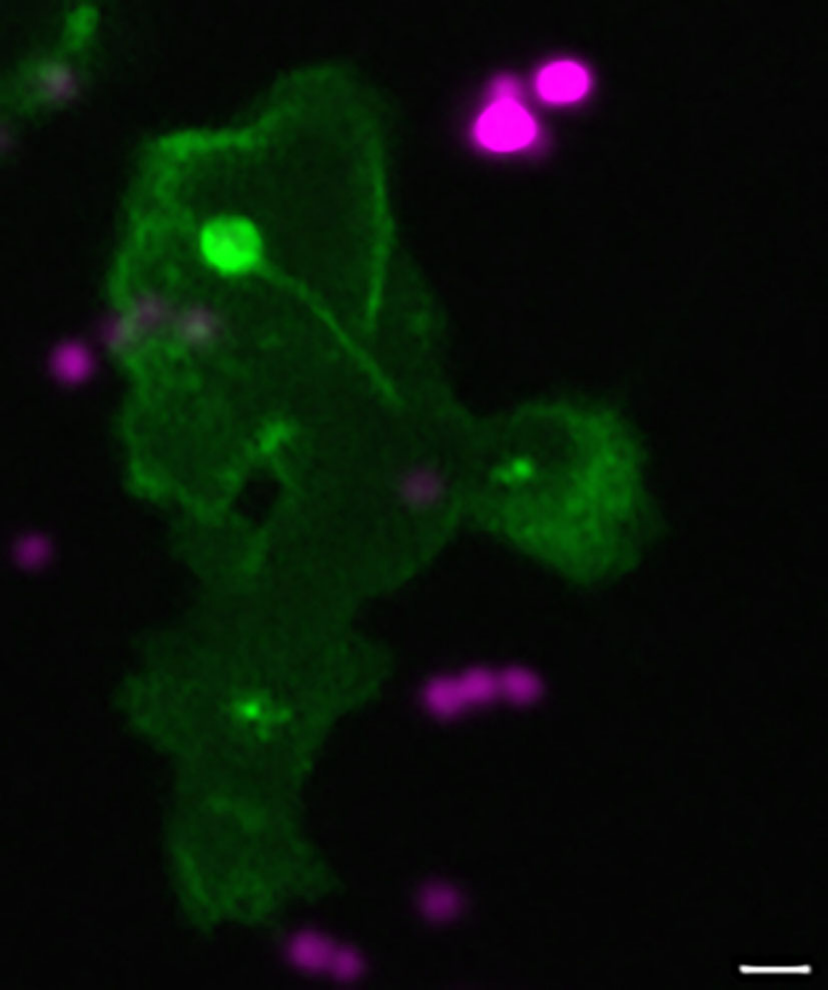
Actin polymerisation around a tether extending from the cell membrane to a phagosome inside a neutrophil recruited to a zebrafish tissue infection site.

**Why did you choose DMM for your paper?**

I chose DMM because it is a high quality journal known for its rigorous and fair review process. The reviews I received were pertinent, thoughtful and really helped to improve my paper. I am delighted to see my article in print with DMM.

Design of group awards would greatly improve the professional lives of other young scientists trying to bridge the gap between postdoc and independence.

**Given your current role, what challenges do you face and what changes could improve the professional lives of other scientists in this role?**

I am a clinician scientist with two young children and it can be difficult to manage commitments to clinical work, research and family life. Nevertheless, I am very well supported at the University of Edinburgh and by my colleagues at the University of Sheffield, with whom I still work. I am now a co-PI on a Wellcome Discovery Award, led by Professor Jason King at the University of Sheffield. Jason designed this award so that I could set up my own lab at the University of Edinburgh and therefore continue the work which we started together during my PhD. This has been a massive help as I have not needed to apply for further funding and I am now part of a 4 lab team for the next 8 years. Design of group awards would greatly improve the professional lives of other young scientists trying to bridge the gap between postdoc and independence. Institute funded technicians with dedicated time to support clinicians would also be of huge help to clinicians trying to juggle clinical/research commitments.

**What's next for you?**

I'm setting up my own lab at the University of Edinburgh. My research will focus on understanding how phagocytes kill and digest different micro-organisms.

**Tell us something interesting about yourself that wouldn't be on your CV**

I really enjoy pond gardening.
